# Supplementary material for: Modeling glioblastoma heterogeneity as a dynamic network of cell states
Source: Mol Syst Biol. 2021 Sep 16;17(9):e10105. doi: 10.15252/msb.202010105 (PMC8444284; doi:10.15252/msb.202010105)
Supplement: Supplementary file 5 — Source Data for Figure 3 [file MSB-17-e10105-s001.zip › Figure3A_sourcedata/GSEA_3065/hallmarks_state4.GseaPreranked.1623416296068/HALLMARK_P53_PATHWAY.html]

Details for gene set HALLMARK\_P53\_PATHWAY[GSEA]

|  || Dataset | state4 |
| Phenotype | NoPhenotypeAvailable |
| Upregulated in class | na\_pos |
| GeneSet | HALLMARK\_P53\_PATHWAY |
| Enrichment Score (ES) | 0.33289033 |
| Normalized Enrichment Score (NES) | 1.4354277 |
| Nominal p-value | 0.003984064 |
| FDR q-value | 0.108167015 |
| FWER p-Value | 0.393 |
Table: GSEA Results Summary

  

Fig 1: Enrichment plot: HALLMARK\_P53\_PATHWAY      
 Profile of the Running ES Score & Positions of GeneSet Members on the Rank Ordered List

  

| PROBE | GENE SYMBOL | GENE\_TITLE | RANK IN GENE LIST | RANK METRIC SCORE | RUNNING ES | CORE ENRICHMENT || 1 | SAT1 |  |  | 4 | 0.669 | 0.0669 | Yes |
| 2 | MXD4 |  |  | 19 | 0.400 | 0.1051 | Yes |
| 3 | ZMAT3 |  |  | 34 | 0.338 | 0.1370 | Yes |
| 4 | FOXO3 |  |  | 72 | 0.273 | 0.1586 | Yes |
| 5 | TXNIP |  |  | 92 | 0.254 | 0.1812 | Yes |
| 6 | RPS27L |  |  | 149 | 0.223 | 0.1947 | Yes |
| 7 | RPL36 |  |  | 246 | 0.186 | 0.1980 | Yes |
| 8 | RPS12 |  |  | 273 | 0.180 | 0.2119 | Yes |
| 9 | CDKN1A |  |  | 319 | 0.171 | 0.2219 | Yes |
| 10 | TSC22D1 |  |  | 369 | 0.160 | 0.2302 | Yes |
| 11 | ABAT |  |  | 435 | 0.145 | 0.2343 | Yes |
| 12 | RACK1 |  |  | 479 | 0.140 | 0.2415 | Yes |
| 13 | DDIT4 |  |  | 493 | 0.138 | 0.2534 | Yes |
| 14 | ZFP36L1 |  |  | 497 | 0.137 | 0.2668 | Yes |
| 15 | BAX |  |  | 521 | 0.134 | 0.2765 | Yes |
| 16 | RPL18 |  |  | 549 | 0.131 | 0.2854 | Yes |
| 17 | NINJ1 |  |  | 582 | 0.127 | 0.2931 | Yes |
| 18 | ISCU |  |  | 608 | 0.125 | 0.3017 | Yes |
| 19 | FAM162A |  |  | 660 | 0.119 | 0.3054 | Yes |
| 20 | ITGB4 |  |  | 680 | 0.117 | 0.3141 | Yes |
| 21 | BTG2 |  |  | 689 | 0.116 | 0.3245 | Yes |
| 22 | HEXIM1 |  |  | 802 | 0.104 | 0.3168 | Yes |
| 23 | S100A10 |  |  | 812 | 0.103 | 0.3258 | Yes |
| 24 | FOS |  |  | 832 | 0.101 | 0.3329 | Yes |
| 25 | PRMT2 |  |  | 1030 | 0.086 | 0.3096 | No |
| 26 | TRIAP1 |  |  | 1240 | 0.074 | 0.2831 | No |
| 27 | RALGDS |  |  | 1366 | 0.068 | 0.2697 | No |
| 28 | TOB1 |  |  | 1439 | 0.065 | 0.2645 | No |
| 29 | MDM2 |  |  | 1450 | 0.064 | 0.2694 | No |
| 30 | CYFIP2 |  |  | 1519 | 0.061 | 0.2645 | No |
| 31 | IP6K2 |  |  | 1813 | 0.052 | 0.2223 | No |
| 32 | WWP1 |  |  | 1818 | 0.052 | 0.2268 | No |
| 33 | HINT1 |  |  | 1908 | 0.049 | 0.2174 | No |
| 34 | MAPKAPK3 |  |  | 2068 | 0.045 | 0.1961 | No |
| 35 | RCHY1 |  |  | 2267 | 0.040 | 0.1680 | No |
| 36 | RAP2B |  |  | 2271 | 0.040 | 0.1715 | No |
| 37 | SESN1 |  |  | 2310 | 0.039 | 0.1693 | No |
| 38 | PHLDA3 |  |  | 2311 | 0.039 | 0.1732 | No |
| 39 | CCND2 |  |  | 2367 | 0.038 | 0.1681 | No |
| 40 | PMM1 |  |  | 2478 | 0.035 | 0.1538 | No |
| 41 | PDGFA |  |  | 2529 | 0.034 | 0.1492 | No |
| 42 | SERTAD3 |  |  | 2564 | 0.034 | 0.1471 | No |
| 43 | MKNK2 |  |  | 2566 | 0.034 | 0.1503 | No |
| 44 | PITPNC1 |  |  | 2624 | 0.032 | 0.1443 | No |
| 45 | ELP1 |  |  | 2663 | 0.031 | 0.1413 | No |
| 46 | RRP8 |  |  | 2679 | 0.031 | 0.1420 | No |
| 47 | VWA5A |  |  | 2702 | 0.031 | 0.1416 | No |
| 48 | CSRNP2 |  |  | 2866 | 0.027 | 0.1179 | No |
| 49 | LDHB |  |  | 2930 | 0.026 | 0.1103 | No |
| 50 | KIF13B |  |  | 2989 | 0.025 | 0.1034 | No |
| 51 | XPC |  |  | 2998 | 0.025 | 0.1046 | No |
| 52 | RXRA |  |  | 3145 | 0.022 | 0.0831 | No |
| 53 | TGFB1 |  |  | 3283 | 0.019 | 0.0628 | No |
| 54 | TRAF4 |  |  | 3337 | 0.017 | 0.0559 | No |
| 55 | PPP1R15A |  |  | 3474 | 0.013 | 0.0352 | No |
| 56 | BAIAP2 |  |  | 3566 | 0.010 | 0.0214 | No |
| 57 | RETSAT |  |  | 3588 | 0.009 | 0.0190 | No |
| 58 | BLCAP |  |  | 3639 | 0.008 | 0.0116 | No |
| 59 | NOL8 |  |  | 3700 | 0.005 | 0.0024 | No |
| 60 | CTSF |  |  | 3820 | -0.000 | -0.0169 | No |
| 61 | EI24 |  |  | 3868 | -0.002 | -0.0243 | No |
| 62 | HRAS |  |  | 3926 | -0.006 | -0.0330 | No |
| 63 | JUN |  |  | 3979 | -0.008 | -0.0406 | No |
| 64 | BTG1 |  |  | 4064 | -0.013 | -0.0529 | No |
| 65 | FUCA1 |  |  | 4070 | -0.013 | -0.0524 | No |
| 66 | ANKRA2 |  |  | 4080 | -0.014 | -0.0524 | No |
| 67 | ABCC5 |  |  | 4102 | -0.015 | -0.0542 | No |
| 68 | DCXR |  |  | 4188 | -0.021 | -0.0659 | No |
| 69 | ATF3 |  |  | 4262 | -0.026 | -0.0751 | No |
| 70 | CTSD |  |  | 4308 | -0.030 | -0.0794 | No |
| 71 | NOTCH1 |  |  | 4321 | -0.031 | -0.0783 | No |
| 72 | F2R |  |  | 4370 | -0.033 | -0.0827 | No |
| 73 | BMP2 |  |  | 4398 | -0.036 | -0.0835 | No |
| 74 | PLK2 |  |  | 4403 | -0.036 | -0.0805 | No |
| 75 | ZNF365 |  |  | 4422 | -0.037 | -0.0796 | No |
| 76 | NUDT15 |  |  | 4571 | -0.049 | -0.0987 | No |
| 77 | CCND3 |  |  | 4578 | -0.050 | -0.0946 | No |
| 78 | NDRG1 |  |  | 4809 | -0.068 | -0.1251 | No |
| 79 | PLXNB2 |  |  | 4962 | -0.080 | -0.1417 | No |
| 80 | EPHA2 |  |  | 5013 | -0.084 | -0.1413 | No |
| 81 | TM7SF3 |  |  | 5039 | -0.086 | -0.1367 | No |
| 82 | TPRKB |  |  | 5099 | -0.092 | -0.1370 | No |
| 83 | PTPN14 |  |  | 5116 | -0.093 | -0.1302 | No |
| 84 | APP |  |  | 5192 | -0.101 | -0.1322 | No |
| 85 | RAD9A |  |  | 5221 | -0.103 | -0.1263 | No |
| 86 | POM121 |  |  | 5294 | -0.109 | -0.1270 | No |
| 87 | GADD45A |  |  | 5297 | -0.109 | -0.1163 | No |
| 88 | PTPRE |  |  | 5306 | -0.110 | -0.1065 | No |
| 89 | SEC61A1 |  |  | 5311 | -0.110 | -0.0960 | No |
| 90 | CD81 |  |  | 5427 | -0.122 | -0.1024 | No |
| 91 | AEN |  |  | 5454 | -0.125 | -0.0939 | No |
| 92 | RAD51C |  |  | 5525 | -0.134 | -0.0917 | No |
| 93 | SLC7A11 |  |  | 5582 | -0.141 | -0.0866 | No |
| 94 | CD82 |  |  | 5621 | -0.147 | -0.0779 | No |
| 95 | STEAP3 |  |  | 5672 | -0.155 | -0.0703 | No |
| 96 | PERP |  |  | 5859 | -0.196 | -0.0807 | No |
| 97 | TM4SF1 |  |  | 5904 | -0.208 | -0.0668 | No |
| 98 | SDC1 |  |  | 5998 | -0.237 | -0.0580 | No |
| 99 | UPP1 |  |  | 6016 | -0.245 | -0.0360 | No |
| 100 | PCNA |  |  | 6173 | -0.374 | -0.0235 | No |
| 101 | SLC3A2 |  |  | 6174 | -0.376 | 0.0144 | No |
Table: GSEA details [plain text format]

  

Fig 2: HALLMARK\_P53\_PATHWAY: Random ES distribution      
 Gene set null distribution of ES for **HALLMARK\_P53\_PATHWAY**
